# Supplementary material for: Translating the potential of the urine steroid metabolome to stage NAFLD (TrUSt-NAFLD): study protocol for a multicentre, prospective validation study
Source: BMJ Open. 2024 Jan 19;14(1):e074918. doi: 10.1136/bmjopen-2023-074918 (PMC10806741; doi:10.1136/bmjopen-2023-074918)
Supplement: Author's manuscript [file bmjopen-2023-074918.draft_revisions.pdf]

BMJ Open is committed to open peer review. As part of this commitment we make the peer review history of every article we publish publicly available.

When an article is published we post the peer reviewers' comments and the authors' responses online. We also post the versions of the paper that were used during peer review. These are the versions that the peer review comments apply to.

The versions of the paper that follow are the versions that were submitted during the peer review process. They are not the versions of record or the final published versions. They should not be cited or distributed as the published version of this manuscript.

BMJ Open is an open access journal and the full, final, typeset and author-corrected version of record of the manuscript is available on our site with no access controls, subscription charges or pay-per-view fees (http://bmjopen.bmj.com).

If you have any questions on BMJ Open's open peer review process please email info.bmjopen@bmj.com

BMJ Open

Translating the potential of the urine steroid metabolome to stage NAFLD (TrUSt NAFLD) – study protocol for a multi-centre, prospective validation study

Journal:	*BMJ Open*
Manuscript ID	bmjopen-2023-074918
Article Type:	Protocol
Date Submitted by the Author:	20-Apr-2023

Complete List of Authors:	Miller, Hamish; OCDEM; Queen Mary University London and Barts Health NHS Trust, Barts Liver Centre<br>Harman, David; Royal Berkshire Hospital NHS Foundation Trust<br>Aithal, Guruprasad; Nottingham University Hospitals NHS Trust and University of Nottingham, NIHR Nottingham Biomedical Research Centre<br>Manousou, Pinelopi; Imperial College London, Department of Metabolism, Digestion and Reproduction<br>Cobbold, Jeremy F.; Oxford University Hospitals NHS Foundation Trust, Oxford Liver Unit; Oxford University, NIHR Oxford Biomedical Research Centre<br>Parker, Richard; Leeds Teaching Hospitals NHS Trust, Leeds Liver Unit<br>Sheridan, David; University of Plymouth, Institute of Translational and Stratified Medicine<br>Newsome, Philip; University Hospitals Birmingham NHS Foundation Trust and the University of Birmingham, National Institute for Health Research Birmingham Biomedical Research Centre<br>Karpe, Fredrik; University of Oxford , Oxford center for Diabetes, Endocrinology and Metabolism<br>Neville, Matthew; OCDEM<br>Arlt, Wiebke; University of Birmingham, Institute of Metabolism and Systems Research; Medical Research Council, London Institute of Medical Sciences, Imperial College Hammersmith Campus<br>Sitch, Alice; University Hospitals Birmingham NHS Foundation Trust and the University of Birmingham, National Institute for Health Research Birmingham Biomedical Research Centre; University of Birmingham, Institute of Applied Health Research<br>Korbonits, Marta; William Harvey Research Institute, Barts and the London School of Medicine and Dentistry, Queen Mary University of London, Centre for Endocrinology<br>Biehl, Michael; University of Groningen, Faculty of Science and Engineering, Bernoulli Institute for Mathematics, Computer Science and Artificial Intelligence; University of Birmingham, SMQB, Institute of Metabolism and Systems Research<br>Alazawi, William; Queen Mary University London and Barts Health NHS Trust, Barts Liver Centre<br>Tomlinson , Jeremy ; OCDEM

Keywords:	Hepatology < INTERNAL MEDICINE, General endocrinology < DIABETES & ENDOCRINOLOGY, DIABETES & ENDOCRINOLOGY

SCHOLARONE™
Manuscripts

Translating the potential of the urine steroid metabolome to stage NAFLD (TrUSt NAFLD) – study protocol for a multi-centre, prospective validation study

Hamish Miller[1 2], David J Harman[3], Guruprasad P. Aithal[4], Pinelopi Manousou[5], Jeremy F. Cobbold[6], Richard Parker[7] , David A Sheridan[8], Philip N Newsome[9], Fredrik Karpe[1], Matthew Neville[1], Wiebke Arlt[10,11], Alice J. Sitch[9,12], Márta Korbonits[13], Michael Biehl[14, 15], William Alazawi[2], Jeremy W Tomlinson[1]

1. Oxford Centre for Diabetes, Endocrinology and Metabolism and NIHR Oxford Biomedical Research Centre, University of Oxford, Oxford, UK.
2. Barts Liver Centre, Queen Mary University London and Barts Health NHS Trust, London, UK
3. Royal Berkshire Hospital NHS Foundation Trust, Reading, UK
4. NIHR Nottingham Biomedical Research Centre, Nottingham University Hospitals NHS Trust and University of Nottingham, Nottingham, UK.
5. Department of Metabolism, Digestion and Reproduction, Imperial College London, London, United Kingdom.
6. Oxford Liver Unit, Oxford University Hospitals NHS Foundation Trust, Oxford, UK; UK NIHR Oxford Biomedical Research Centre, University of Oxford, Oxford, UK
7. Leeds Liver Unit, Leeds Teaching Hospitals NHS Trust, Leeds, UK.
8. Institute of Translational and Stratified Medicine, University of Plymouth, Plymouth, UK
9. National Institute for Health Research Birmingham Biomedical Research Centre at University Hospitals Birmingham NHS Foundation Trust and the University of Birmingham, Birmingham, UK.
10. Institute of Metabolism and Systems Research, University of Birmingham, Edgbaston, Birmingham, UK. Centre for Endocrinology, Diabetes and Metabolism, Birmingham Health Partners, University Hospitals Birmingham NHS Foundation Trust, Edgbaston, Birmingham, UK.
11. Medical Research Council London Institute of Medical Sciences, Imperial College Hammersmith Campus, London, UK.
12. Institute of Applied Health Research, University of Birmingham, and NIHR Birmingham Biomedical Research Centre, University of Birmingham and University Hospitals Birmingham NHS Foundation Trust, Birmingham, United Kingdom.
13. Centre for Endocrinology, William Harvey Research Institute, Barts and the London School of Medicine and Dentistry, Queen Mary University of London, London, UK

For peer review only

14. Bernoulli Institute for Mathematics, Computer Science and Artificial Intelligence, Faculty of Mathematics and Natural Sciences, University of Groningen, Groningen, The Netherlands

15. SMQB, Institute of Metabolism and Systems Research, College of Medical and Dental Sciences, University of Birmingham, Birmingham, United Kingdom

Email Adresses

Hamish Miller: hamish.miller@nhs.net

David J Harman: david.harman@royalberkshire.nhs.uk

Guruprasad P. Aithal: guru.aithal@nottingham.ac.uk

Pinelopi Manousou: pinelopi.manousou@nhs.net

Jeremy F. Cobbold: jeremy.Cobbold@ouh.nhs.uk

Richard Parker: richardparker@nhs.net

David A Sheridan: david.sheridan2@nhs.net

Philip N Newsome: p.n.newsome@bham.ac.uk

Fredrik Karpe: fredrik.karpe@ocdem.ox.ac.uk

Matthew Neville: matthew.neville@ocdem.ox.ac.uk

Wiebke Arlt: w.arlt@lms.mrc.ac.uk

Alice J. Sitch: a.j.sitch@bham.ac.uk

Márta Korbonits: m.korbonits@qmul.ac.uk

Michael Biehl: m.biehl@rug.nl

William Alazawi: w.alazawi@qmul.ac.uk

Jeremy W Tomlinson: jeremy.tomlinson@ocdem.ox.ac.uk

Corresponding Author:

Prof Jeremy Tomlinson

Oxford Centre for Diabetes, Endocrinology & Metabolism

University of Oxford

Churchill Hospital

Headington

Oxford

OX3 7LJ

Email: jeremy.tomlinson@ocdem.ox.ac.uk

Tel. 01865 857359

For peer review only

Abstract

Introduction

Non-alcoholic fatty liver disease (NAFLD) affects approximately 1 in 4 individuals and its prevalence continues to rise. The advanced stages of NAFLD with significant liver fibrosis are associated with adverse morbidity and mortality outcomes. Currently, liver biopsy remains the 'gold-standard' approach to stage NAFLD severity. Although generally well tolerated, liver biopsies are associated with significant complications, are resource intensive, costly, sample only a very small area of the liver and require day case admission to a secondary care setting. As a result, there is a significant unmet need to develop non-invasive biomarkers that can accurately stage NAFLD and limit the need for liver biopsy. The aim of this study is to validate the use of the urine steroid metabolome as a strategy to stage NAFLD severity and to compare its performance against other non-invasive NAFLD biomarkers.

Methods and Analysis

The TrUSt NAFLD study is a multi-centre prospective test validation study aiming to recruit 310 patients with biopsy-proven and staged NAFLD across 8 centres within the UK. 150 appropriately matched control patients without liver disease will be recruited through the Oxford Biobank. Blood and urine samples, alongside clinical data, will be collected from all participants. Urine samples will be analysed by liquid chromatography-tandem mass spectroscopy to quantify a panel of pre-defined steroid metabolites. A machine learning based classifier, e.g. Generalized Matrix Relevance Learning Vector Quantization (GMLVQ) that was trained on retrospective samples, will be applied to the prospective steroid metabolite data to determine its ability to identify those patients with advanced, as opposed to mild-moderate, liver fibrosis as a consequence of NAFLD.

Ethics and dissemination

Research ethical approval was granted by West Midlands - Black Country Research Ethics Committee (REC reference: 21/WM/0177). A substantial amendment (TrUSt-NAFLD-SA1) was approved on 26/11/21.

Registration details

Title: Translating the potential of the urine steroid metabolome to stage non-alcoholic fatty liver disease

Trial ID: ISRCTN19370855

Date registered: 05/01/2023

For peer review only

Article summary

Strengths and limitations of this study:

Strengths

- The study aims to prospectively validate a non-invasive urine test which could easily be incorporated as part of a screening to identify individuals at high risk of advanced NAFLD.
- The participants recruited with NAFLD patients will all have had a liver biopsy to stage their disease severity as well as multiple other non-invasive biomarkers that are currently used in clinical practice.
- The study includes a well-matched and phenotyped control cohort without evidence of liver disease, prospectively recruited from an existing biobank.

Limitations

- The study is cross-sectional and not longitudinal, so will not be able to address questions as to the utility of biomarker strategies and disease progression / remission.
- The study will recruit patients having a liver biopsy as part of routine clinical care. This may introduce a degree of selection bias, creating a cohort more likely to have advanced (as opposed to mild) disease severity.

Keywords

Non alcoholic fatty liver disease

urine steroid metabolome

biomarkers

For peer review only

Introduction

Non-alcoholic fatty liver disease (NAFLD) is the most prevalent chronic liver condition [1]. It affects up to 30% of the population (rising to 60% of patients with diabetes and obesity); >50 million adults in Europe and >10 million adults in the UK. It is the second commonest indication of liver transplantation worldwide [2, 3] and now accounts for 8.4% of liver transplants in Europe [4]. The advanced stages are associated with a 4-5-fold increase in mortality and morbidity, driven not only through liver-specific causes (including hepatocellular carcinoma), but through cardiovascular events [5, 6]. Estimates suggest that NAFLD costs the UK in excess of £5 billion per year and, globally, the expected 10-year burden of NAFLD could exceed $1.005 trillion in the USA and €334 billion in Europe [7].

NAFLD is a spectrum of disease ranging from simple steatosis through to inflammation (non-alcoholic steatohepatitis, NASH) to fibrosis (graded from F0-F4) with the risk of cirrhosis (F4) and the development of hepatocellular carcinoma (HCC). Fibrosis stage is tightly associated with an adverse clinical outcome with both liver-specific and cardiovascular morbidity and mortality [8, 9] The current reference standard for assessment of NAFLD severity is liver biopsy; however, there are significant complications and poor patient acceptability [10, 11]. In addition, it is a resource intensive and costly procedure, requires day case admission and time off work for the patient, samples a very small area of the liver and the interpretation of the biopsy findings are fraught with inter-individual variability [12]. Therefore, the development of accurate, non-invasive markers to stage NAFLD is a critical unmet priority clinical need. Routine interpretation of liver biochemistry is unhelpful; 50% of patients with advanced fibrosis (F3 or F4) have normal liver chemistry [13]. Several non-invasive tools (serological, clinical, imaging) have been developed; however, to date, none have been shown to be sufficiently robust to replace liver biopsy [14].

The liver is the main site of steroid metabolism and there is disruption of specific pathways in patients with NAFLD [15, 16], providing biological plausibility to our approach. The pilot study showed an increased activity in 11β-hydroxysteroid dehydrogenase type 1 (11BHSD1) and 5α-reductase in patients with advanced fibrosis [17]. Steroid 5β- reductase (AKR1D1), which is mostly expressed in the liver, has also been shown to decrease as fibrosis in NAFLD progresses [18]. When coupled with machine learning-based analysis using, for instance, Generalized Matrix Learning Vector Quantization (GMLVQ), a global assessment of hepatic steroid hormone metabolism can be made. Data from our published proof-of-principle study indicate that this is tightly influenced by NAFLD stage [17]. As part of this discovery study, we analysed urine samples from 227 subjects (121 with biopsy-proven NAFLD and 106 controls).

For peer review only

GMLVQ analysis achieved excellent separation of early (F0-2) from advanced (F3-4) fibrosis (AUC ROC: 0.92 [0.91-0.94]). There was near perfect separation of healthy controls from patients with advanced fibrotic NAFLD and from those with NAFLD cirrhosis. Further refinement of the model incorporated the 10 most discriminatory steroids as well as patient age and BMI which has further enhanced diagnostic performance [17].

The aim of the current study is to prospectively validate our published findings in an independent cohort of patients undergoing routine liver biopsy for NAFLD and to compare the performance of the machine learning based urine steroid metabolome test against other non-invasive biomarker strategies that are currently used in clinical practice.

For peer review only

Methods and analysis

Design

TrUSt-NAFLD is a prospective, observational, non-interventional clinical study where recruitment and participation is designed to coincide with routine National Health Service (NHS) clinical care aiming to avoid the need for additional venepuncture, minimise inconvenience to participants and maximize recruitment potential. Occasionally, additional study specific visits for participants will be scheduled where this can facilitate recruitment or ensure that samples are collected within the pre-defined time limitations of the study (samples collected within 12-months of liver biopsy).

A dedicated control cohort of participants without evidence of liver disease will be prospectively recruited from an existing biobank of patients (Oxford Biobank (OBB), https://www.oxfordbiobank.org.uk) who have undergone detailed metabolic phenotyping and provided consent to be recalled by phenotype for additional clinical studies. These participants will not undergo liver biopsies but will have a comprehensive assessment of non-invasive NAFLD biomarkers measured as well as urine steroid metabolite analysis.

Setting and participants

The study will take place in secondary care hospital settings in 8 dedicated hepatology clinics across the UK. Patients (male or female), aged ≥18 years, who are attending or have attended an NHS clinic for the diagnosis and / or management of NAFLD and who are scheduled to have a liver biopsy or who have had a liver biopsy to stage NAFLD within the last 12 months will be approached to participate. Control participants without evidence of NAFLD will be recruited from the Oxford Biobank based at the Oxford Centre for Endocrinology, Diabetes and Metabolism, Churchill Hospital Oxford, UK.

Patient and public involvement

The study protocol and associated supporting documentation was reviewed by a dedicated patient and public involvement panel that is supported by the National Institute for Health and Care Research (NIHR) Oxford Biomedical Research Centre (BRC) and their feedback was incorporated into the final study documents. The panel will continue to be involved with dissemination activities following study completion.

Recruitment

The TrUSt-NAFLD study will recruit patients with a diagnosis of NAFLD from dedicated NHS hepatology clinics. The initial approach for participants to be part of the study will be made by

For peer review only

members of the team directly involved in their clinical care. In some situations, an alternative recruitment strategy whereby patients (who have consented to be approached for future research) are contacted having been identified from existing clinical databases may be employed. To ensure sufficient recruitment of patients, 8 centres across the UK will participate in the study. Interested potential participants will be seen during their routine NHS clinic visit by the research team to confirm eligibility. Those patients identified from existing databases will be contacted by letter and their eligibility will have been assessed prior to contact by lead clinicians and researchers within the dedicated hepatology team.

Recruitment of control participants without NAFLD will utilize the OBB; a biobank of >9000 volunteers in Oxfordshire, UK who have undergone extensive metabolic phenotyping and consented to be re-approached for clinical research. In order to try to ensure the absence of significant liver disease, individuals with a HOMA-IR (as a marker of global insulin resistance) https://www.dtu.ox.ac.uk/homacalculator/ in the lowest 25%, body mass index (BMI) <35kg/m^2 and fasting triglyceride levels <2 mmol/l will be invited to participate. HOMA-IR, triglycerides and BMI are all measured and recorded as part of the detailed OBB metabolic phenotyping and therefore can be used in a recall-by-phenotype strategy. Invitations to take part in the study will be sent with an aim of recruiting 60% male and 40% female participants to match the anticipated sex distribution in the NAFLD cohort which has been estimated from local liver biopsy data. Only individuals with normal liver biochemistry, and non-invasive serum markers (including the enhanced liver fibrosis panel, ELF) indicating low-risk of advanced NAFLD will be included in the study

Screening and eligibility assessment

For potential participants with a diagnosis of NAFLD, screening will be undertaken by the clinical care team through direct confirmation of age, establishing that there is a diagnosis of NAFLD and that they either have a liver biopsy scheduled or have had a liver biopsy within the last 12-months. Potential participants will be provided with a letter of invitation and a patient information leaflet (PIL) either at the time of their routine NHS clinic visit or sent by post. For potential control participants without NAFLD, screening of the existing OBB database will be undertaken and a letter of invitation and PIL will be sent by members of the OBB research team.

Specific inclusion and exclusion criteria are detailed below. There will be no exceptions made regarding eligibility and each participant must satisfy all the approved inclusion and exclusion criteria of the protocol.

For peer review only

Inclusion Criteria

Participants with NAFLD

- Participant is willing and able to give informed consent for participation in the study.
- Patients with a diagnosis of NAFLD who are scheduled for a liver biopsy, or the patient has had a liver biopsy with a confirmed diagnosis of NAFLD within the last 12 months.
- Aged ≥18 years.

Control participants without evidence of NAFLD

- Participant is willing and able to give informed consent for participation in the study.
- Bottom 25th percentile of HOMA-IR in Oxford Biobank.
- Body mass index (BMI) <35kg/m$^{2.}$
- Fasting triglycerides<2.0mmol/L.
- Aged ≥18 years.

Exclusion Criteria

All participants

- Insufficient understanding of written and verbal English.
- The participant has not had sufficient time to read the PIL and understand the study requirements.
- Hepatic steatosis, inflammation, or fibrosis with a primary aetiology other than NAFLD.

Study procedures

All participants will personally sign and date the latest approved version of the informed consent form before any study specific procedures are performed. Written and verbal versions of the PIL and informed consent forms will be presented to the participants detailing the exact nature of the study, what it will involve for the participant, the implications and constraints of the protocol, and any risks involved in taking part. It will be clearly stated that the participant is free to withdraw from the study at any time for any reason without prejudice to their future care, without affecting their legal rights, and with no obligation to give the reason for withdrawal. The participant will be allowed as much time as possible (and this may be less

For peer review only

than 24-hours) to consider the information, and the opportunity to question the investigators, their general practitioner or other independent parties to decide whether they will participate in the study. Consent may be obtained and the study procedures performed on the same day. The person who obtains the informed consent will be suitably qualified, experienced and have been authorised to do so by the chief/principal investigator. A copy of the signed informed consent form will be given to the participant. The original signed form will be retained at the study site. The study is not randomised. Participants will be enrolled as they are consented. At enrolment, all participants will be assigned a unique identification number in a sequential order.

For all participants, demographic data will be collected including age, sex, ethnicity, height, weight and calculated BMI. Details of the participants'participant's clinical history will also be collected, including any relevant past medical history (smoking, type 2 diabetes, hypertension, cardiovascular disease and alcohol use) as well as details of concomitant prescribed medications. Patients with NAFLD will usually have routine blood tests as part of their clinical care consultation and therefore the additional blood samples that will be collected will not require additional venepuncture (although this may be needed if a participant attends for a dedicated research visit that is separate from their clinical care appointment). A dedicated research visit is required for all participants without NAFLD that will include collecting demographic data and clinical history as well as venepuncture and providing a 10mL urine sample. Vibration-controlled transient elastography (VCTE) is frequently used in hepatology clinics to determine the risk of advanced fibrosis and can provide both a measure of liver stiffness (indicative of fibrosis stage) and a controlled attenuation parameter (CAP) that correlates with the degree of hepatic steatosis [19, 20]. Where available, these data will be recorded for all patients with NAFLD.

All blood sample analysis will include measurement of a full blood count, renal function and electrolytes, liver chemistry (including alanine aminotransaminase (ALT), aspartate aminotransaminase (AST), alkaline phosphatase (ALP), gamma-glutamyltransferase (GGT), bilirubin, albumin, ferritin), total and HDL-cholesterol and triglycerides as well as glycated haemoglobin. In addition, samples will be analysed for the Enhanced Liver Fibrosis (ELF) panel [21] (hyaluronic acid, type III procollagen peptide, and tissue inhibitor of matrix metalloproteinase 1). Calculations for non-invasive risk score to detect advanced fibrosis will also be made including NAFLD fibrosis score (NFS), Fib-4, APRI, Fibroscan-AST (FAST) and BARD [22-26].

Liver biopsy data will be collected to include the NAFLD activity score (NAS) grading with its individual components of steatosis, lobular inflammation, and ballooning and also the fibrosis staging (graded F0-F4) according to the Kleiner classification [27].

For peer review only

A 10mL urine sample will be collected from all participants, aliquoted and stored at -80°C until analysis. Pre-defined steroid hormone metabolites will be measured using gas chromatography mass spectrometry (GC-MS) and liquid chromatography tandem mass spectrometry (LC MS-MS) as described previously [28] [29]. The steroid metabolite data will then be subjected to machine learning employing a GMLVQ [17] classifier that was trained on retrospective data. Its performance to identify participants with advanced fibrotic NAFLD compared against other non-invasive biomarker strategies (Fib-4, NFS, BARD, APRI, AST/ALT, ELF, FAST-AST, VCTE) will be evaluated.

Statistical methods, sample size and analysis plan

The study has been planned and designed with expert statistical advice and an experienced statistician is part of the investigative team.

Sample size

We will aim to recruit at least 460 participants to the TrUSt-NAFLD study (NAFLD n=310; control n=150). Sample size and power calculations have been based upon our previous published data [17]. Our primary analysis will aim to distinguish patients with mild (F0-2) *vs.* advanced fibrosis (F3-4). Through the recruitment of 310 participants with NAFLD, we will have 85% power to identify a difference in sensitivity between technologies of 12 percentage points (80% vs 92%) assuming a 5% significance level, a prevalence of 50% (i.e. 50% of the participants will be F0-2 and 50% F3-4) and independence of comparative test results. The estimates of sensitivity and the difference in sensitivity have been estimated from previous work [14].

Analysis of Outcome Measures

Data for all participants who complete the study will be included in the analysis. If any participants withdraw from the study, samples and data collected will not routinely be destroyed and will be used in the analysis unless requested not to do so by the individual. Statistical analysis will be undertaken at the end of study and after the analysis of all the samples has been completed.

The primary outcome of the TrUSt-NAFLD study is to determine the accuracy of the urine steroid metabolome GMLVQ algorithm to distinguish early (F0-2) from advanced (F3-4) NAFLD fibrosis. Sensitivity, specificity, negative and positive predictive values will be

For peer review only

calculated with 95% confidence intervals. Additionally, receiver operating characteristic curves will be generated and the area under the curve will be calculated.

Secondary and exploratory outcomes will include a comparison of the performance of the urine steroid metabolome GMLVQ algorithm against other non-invasive NAFLD biomarkers (Fib-4, NFS, BARD, APRI, AST/ALT, ELF, FAST-AST, VCTE). Data from participants with NAFLD will also be compared against control participants without evidence of NAFLD to determine the ability of the algorithm to detect NAFLD (across the spectrum of its severity) in the general population. Finally, exploratory analyses detecting steroid metabolites using LC MS-MS in serum samples and subsequently incorporating the GMLVQ algorithm will be performed and compared against the urine analysis.

For peer review only

Ethics and dissemination

Ethical and safety considerations

The chief investigator will ensure that this study is conducted in accordance with the principles of the Declaration of Helsinki and that the study is conducted in accordance with relevant regulations and with Good Clinical Practice.

The study will not pose significant ethical issues or risks to the participants. Sample collection and study visits for those individuals with NAFLD will, in the majority of cases, coincide with routine clinical care appointments, and so additional venepuncture, and its associated discomfort, will not be needed. Providing the urine sample provides no additional risk. Where additional study appointments are required, travel expenses are reimbursed and a small payment to cover inconvenience will be offered. Incidental abnormal finding unrelated to the study will be discussed with the participants and where appropriate, and with their consent, referral to their general practitioner or suitable specialist will be made.

The TrUSt-NAFLD study will be sponsored by University of Oxford, UK. All relevant study supporting documentation as well as the clinical protocol have been reviewed and approved by West Midlands, Black Country Research Ethics Committee (REC) (ref. 21/WM/0177) and the Health Research Authority (HRA) (https://www.hra.nhs.uk).

The OBB is sponsored by the University of Oxford, UK and supported by the NIHR Oxford BRC. The protocols and supporting documentation for recruitment, investigations and recruiting by phenotype and genotype have been reviewed and approved by South Central, Oxford C NHS REC (ref. 18/SC/0588) and the HRA. An annual progress report will be submitted to the REC and HRA (where required) host organisation and sponsor. In addition, on study completion, an End of Study notification and final report will be submitted to the same parties.

Transparency in research

Details of the study will be made available on HRA research summaries https://www.hra.nhs.uk/planning-and-improving-research/application-summaries/research-summaries/. The study details will also be available on University of Oxford and Radcliffe Department of Medicine websites. The study is registered on the International Standard Registered Clinical/soCial sTudy Number (ISRCTN) database (ISRCTN19370855, https://doi.org/10.1186/ISRCTN19370855).

For peer review only

Data statement

The datasets generated during and/or analysed during the current study will be stored in a non-publicly available repository

Dissemination plan (publications, data deposition and curation)

A copy of the final report and / or publication will be sent to study participants upon request. Results will be disseminated through presentations at national and international conferences and publications sent to peer-review journals with open access in accordance with the funder's publication policy (https://wellcome.org/grant-funding/guidance/open-access-guidance/open-access-policy). In addition, research findings will be disseminated through dedicated departmental public engagement events and through patient support groups.

The investigators will be involved in reviewing drafts of the manuscripts, abstracts, press releases and any other publications arising from the study. Authors will acknowledge that the study was funded by Wellcome. Authorship will be determined in accordance with the ICMJE guidelines and other contributors will be acknowledged.

Patient and Public Involvement

The protocol and proposed plan of investigations was reviewed by the Patient and Public Involvement and Engagement (PPIE) panel that is supported but the NIHR Oxford Biomedical Centre. All panel members provided positive feedback and expressed a preference for the potential of providing a urine sample over the need for a liver biopsy. On completion, data from the study will be presented to the panel who will be actively involved in public engagement and dissemination activities.

For peer review only

Author contributions

HM will co-ordinate and run the TrUSt NAFLD study and drafted the manuscript. JWT is the chief investigator for the study and was responsible for the original concept and design of the study. JWT edited the manuscript and was responsible for the final approval of the manuscript. All authors read, contributed to editing and approved the final manuscript.

Funding statement

This study is funded through a Wellcome Innovator Award to JWT, MB and WA (ref: 222627/Z/21/Z) and is supported by the NIHR Oxford Biomedical Research Centre and by the NIHR Birmingham Biomedical Research Centre. The views expressed are those of the authors and not necessarily those of the NIHR or the Department of Health and Social Care.

Competing interest statement

JWT has been an advisory board member for Novo Nordisk, Pfizer and Poxel and has been part of a data and safety monitoring committee for Novartis. RP has been an advisory board member for Novo Nordisk.

A patent related to the proposed work has been filed (GB Patent Application Number 2005435.9, filed 14/4/20, European Patent Application Number: EP21703541.9A, filed 29/1/21 and Worldwide Patent Application Number: WO2021152330A1, filed 29/1/21)

Word count = 3222

For peer review only

References

1. Paik, J.M., et al., *Changes in the Global Burden of Chronic Liver Diseases From 2012 to 2017: The Growing Impact of NAFLD.* Hepatology, 2020. **72**(5): p. 1605-1616.
2. Wong, R.J., et al., *Nonalcoholic steatohepatitis is the second leading etiology of liver disease among adults awaiting liver transplantation in the United States.* Gastroenterology, 2015. **148**(3): p. 547-55.
3. Dulai, P.S., et al., *Increased risk of mortality by fibrosis stage in nonalcoholic fatty liver disease: Systematic review and meta-analysis.* Hepatology, 2017. **65**(5): p. 1557-1565.
4. Haldar, D., et al., *Outcomes of liver transplantation for non-alcoholic steatohepatitis: A European Liver Transplant Registry study.* J Hepatol, 2019. **71**(2): p. 313-322.
5. Singal, A.G., P. Lampertico, and P. Nahon, *Epidemiology and surveillance for hepatocellular carcinoma: New trends.* J Hepatol, 2020. **72**(2): p. 250-261.
6. Younossi, Z.M., et al., *Global epidemiology of nonalcoholic fatty liver disease-Meta-analytic assessment of prevalence, incidence, and outcomes.* Hepatology, 2016. **64**(1): p. 73-84.
7. Younossi, Z.M., et al., *The economic and clinical burden of nonalcoholic fatty liver disease in the United States and Europe.* Hepatology, 2016. **64**(5): p. 1577-1586.
8. Taylor, R.S., et al., *Association Between Fibrosis Stage and Outcomes of Patients With Nonalcoholic Fatty Liver Disease: A Systematic Review and Meta-Analysis.* Gastroenterology, 2020. **158**(6): p. 1611-1625.e12.
9. Sanyal, A.J., et al., *Prospective Study of Outcomes in Adults with Nonalcoholic Fatty Liver Disease.* New England Journal of Medicine, 2021. **385**(17): p. 1559-1569.
10. Neuberger, J., et al., *Guidelines on the use of liver biopsy in clinical practice from the British Society of Gastroenterology, the Royal College of Radiologists and the Royal College of Pathology.* Gut, 2020. **69**(8): p. 1382.
11. Howlett, D.C., et al., *Findings of the UK national audit evaluating image-guided or image-assisted liver biopsy. Part II. Minor and major complications and procedure-related mortality.* Radiology, 2013. **266**(1): p. 226-35.
12. Brunt, E.M., et al., *Complexity of ballooned hepatocyte feature recognition: Defining a training atlas for artificial intelligence-based imaging in NAFLD.* Journal of Hepatology, 2022. **76**(5): p. 1030-1041.
13. Verma, S., et al., *Predictive value of ALT levels for non-alcoholic steatohepatitis (NASH) and advanced fibrosis in non-alcoholic fatty liver disease (NAFLD).* Liver International, 2013. **33**(9): p. 1398-1405.
14. Chin, J.L., et al., *Non-invasive Markers of Liver Fibrosis: Adjuncts or Alternatives to Liver Biopsy?* Front Pharmacol, 2016. **7**: p. 159.
15. Ahmed, A., et al., *A Switch in Hepatic Cortisol Metabolism across the Spectrum of Non Alcoholic Fatty Liver Disease.* PLoS ONE, 2012. **7**(2): p. e29531.
16. Nikolaou, N., et al., *AKR1D1 is a novel regulator of metabolic phenotype in human hepatocytes and is dysregulated in non-alcoholic fatty liver disease.* Metabolism, 2019. **99**: p. 67-80.
17. Moolla, A., et al., *Accurate non-invasive diagnosis and staging of non-alcoholic fatty liver disease using the urinary steroid metabolome.* Alimentary Pharmacology & Therapeutics, 2020. **51**(11): p. 1188-1197.

For peer review only

18. Nikolaou, N., et al., *AKR1D1 is a novel regulator of metabolic phenotype in human hepatocytes and is dysregulated in non-alcoholic fatty liver disease.* Metabolism, 2019. **99**: p. 67-80.
19. Siddiqui, M.S., et al., *Vibration-Controlled Transient Elastography to Assess Fibrosis and Steatosis in Patients With Nonalcoholic Fatty Liver Disease.* Clinical Gastroenterology and Hepatology, 2019. **17**(1): p. 156-163.e2.
20. Pu, K., et al., *Diagnostic accuracy of controlled attenuation parameter (CAP) as a non-invasive test for steatosis in suspected non-alcoholic fatty liver disease: a systematic review and meta-analysis.* BMC Gastroenterology, 2019. **19**(1).
21. Guha, I.N., et al., *Noninvasive markers of fibrosis in nonalcoholic fatty liver disease: Validating the European Liver Fibrosis Panel and exploring simple markers.* Hepatology, 2007. **47**(2): p. 455-460.
22. Angulo, P., et al., *The NAFLD fibrosis score: A noninvasive system that identifies liver fibrosis in patients with NAFLD.* Hepatology, 2007. **45**(4): p. 846-854.
23. Sterling, R.K., et al., *Development of a simple noninvasive index to predict significant fibrosis in patients with HIV/HCV coinfection.* Hepatology, 2006. **43**(6): p. 1317-1325.
24. Wai, C., *A simple noninvasive index can predict both significant fibrosis and cirrhosis in patients with chronic hepatitis C.* Hepatology, 2003. **38**(2): p. 518-526.
25. Newsome, P.N., et al., *FibroScan-AST (FAST) score for the non-invasive identification of patients with non-alcoholic steatohepatitis with significant activity and fibrosis: a prospective derivation and global validation study.* The Lancet Gastroenterology and Hepatology, 2020. **5(4)**: p. 362-373.
26. Harrison, S.A., et al., *Development and validation of a simple NAFLD clinical scoring system for identifying patients without advanced disease.* Gut, 2008. **57**(10): p. 1441-7.
27. Kleiner, D.E., et al., *Design and validation of a histological scoring system for nonalcoholic fatty liver disease.* Hepatology, 2005. **41**(6): p. 1313-1321.
28. Arlt, W., et al., *Urine Steroid Metabolomics as a Biomarker Tool for Detecting Malignancy in Adrenal Tumors.* The Journal of Clinical Endocrinology & Metabolism, 2011. **96**(12): p. 3775-3784.
29. Bancos, I., et al., *Urine steroid metabolomics for the differential diagnosis of adrenal incidentalomas in the EURINE-ACT study: a prospective test validation study.* Lancet Diabetes Endocrinol, 2020. **8**(9): p. 773-781.

For peer review only

BMJ Open

Translating the potential of the urine steroid metabolome to stage NAFLD (TrUSt NAFLD) – study protocol for a multi-centre, prospective validation study

Journal:	*BMJ Open*
Manuscript ID	bmjopen-2023-074918.R1
Article Type:	Protocol
Date Submitted by the Author:	23-Nov-2023
Complete List of Authors:	Miller, Hamish; OCDEM; Queen Mary University London and Barts Health NHS Trust, Barts Liver Centre<br>Harman, David; Royal Berkshire Hospital NHS Foundation Trust<br>Aithal, Guruprasad; Nottingham University Hospitals NHS Trust and University of Nottingham, NIHR Nottingham Biomedical Research Centre<br>Manousou, Pinelopi; Imperial College London, Department of Metabolism, Digestion and Reproduction<br>Cobbold, Jeremy F.; Oxford University Hospitals NHS Foundation Trust, Oxford Liver Unit; Oxford University, NIHR Oxford Biomedical Research Centre<br>Parker, Richard; Leeds Teaching Hospitals NHS Trust, Leeds Liver Unit<br>Sheridan, David; University of Plymouth, Institute of Translational and Stratified Medicine<br>Newsome, Philip; University Hospitals Birmingham NHS Foundation Trust and the University of Birmingham, National Institute for Health Research Birmingham Biomedical Research Centre<br>Karpe, Fredrik; University of Oxford , Oxford center for Diabetes, Endocrinology and Metabolism<br>Neville, Matthew; OCDEM<br>Arlt, Wiebke; University of Birmingham, Institute of Metabolism and Systems Research; Medical Research Council, London Institute of Medical Sciences, Imperial College Hammersmith Campus<br>Sitch, Alice; University Hospitals Birmingham NHS Foundation Trust and the University of Birmingham, National Institute for Health Research Birmingham Biomedical Research Centre; University of Birmingham, Institute of Applied Health Research<br>Korbonits, Marta; William Harvey Research Institute, Barts and the London School of Medicine and Dentistry, Queen Mary University of London, Centre for Endocrinology<br>Biehl, Michael; University of Groningen, Faculty of Science and Engineering, Bernoulli Institute for Mathematics, Computer Science and Artificial Intelligence; University of Birmingham, SMQB, Institute of Metabolism and Systems Research<br>Alazawi, William; Queen Mary University London and Barts Health NHS Trust, Barts Liver Centre<br>Tomlinson , Jeremy ; OCDEM
**Primary Subject Heading**:	Gastroenterology and hepatology

Secondary Subject Heading:	Diagnostics
Keywords:	Hepatology < INTERNAL MEDICINE, General endocrinology < DIABETES & ENDOCRINOLOGY, DIABETES & ENDOCRINOLOGY

SCHOLARONE™
Manuscripts

Translating the potential of the urine steroid metabolome to stage NAFLD (TrUSt NAFLD) – study protocol for a multi-centre, prospective validation study

Hamish Miller[1 2], David J Harman[3], Guruprasad P. Aithal[4], Pinelopi Manousou[5], Jeremy F. Cobbold[6], Richard Parker[7] , David A Sheridan[8], Philip N Newsome[9], Fredrik Karpe[1], Matthew Neville[1], Wiebke Arlt[10,11], Alice J. Sitch[9,12], Márta Korbonits[13], Michael Biehl[14, 15], William Alazawi[2], Jeremy W Tomlinson[1]

1. Oxford Centre for Diabetes, Endocrinology and Metabolism and NIHR Oxford Biomedical Research Centre, University of Oxford, Oxford, UK.
2. Barts Liver Centre, Queen Mary University London and Barts Health NHS Trust, London, UK
3. Royal Berkshire Hospital NHS Foundation Trust, Reading, UK
4. NIHR Nottingham Biomedical Research Centre, Nottingham University Hospitals NHS Trust and University of Nottingham, Nottingham, UK.
5. Department of Metabolism, Digestion and Reproduction, Imperial College London, London, United Kingdom.
6. Oxford Liver Unit, Oxford University Hospitals NHS Foundation Trust, Oxford, UK; UK NIHR Oxford Biomedical Research Centre, University of Oxford, Oxford, UK
7. Leeds Liver Unit, Leeds Teaching Hospitals NHS Trust, Leeds, UK.
8. Institute of Translational and Stratified Medicine, University of Plymouth, Plymouth, UK
9. National Institute for Health Research Birmingham Biomedical Research Centre at University Hospitals Birmingham NHS Foundation Trust and the University of Birmingham, Birmingham, UK.
10. Institute of Metabolism and Systems Research, University of Birmingham, Edgbaston, Birmingham, UK. Centre for Endocrinology, Diabetes and Metabolism, Birmingham Health Partners, University Hospitals Birmingham NHS Foundation Trust, Edgbaston, Birmingham, UK.
11. Medical Research Council London Institute of Medical Sciences, Imperial College Hammersmith Campus, London, UK.
12. Institute of Applied Health Research, University of Birmingham, and NIHR Birmingham Biomedical Research Centre, University of Birmingham and University Hospitals Birmingham NHS Foundation Trust, Birmingham, United Kingdom.
13. Centre for Endocrinology, William Harvey Research Institute, Barts and the London School of Medicine and Dentistry, Queen Mary University of London, London, UK

For peer review only

14. Bernoulli Institute for Mathematics, Computer Science and Artificial Intelligence, Faculty of Mathematics and Natural Sciences, University of Groningen, Groningen, The Netherlands

15. SMQB, Institute of Metabolism and Systems Research, College of Medical and Dental Sciences, University of Birmingham, Birmingham, United Kingdom

Email Addresses:
Hamish Miller: hamish.miller@nhs.net
David J Harman: david.harman@royalberkshire.nhs.uk
Guruprasad P. Aithal: guru.aithal@nottingham.ac.uk
Pinelopi Manousou: pinelopi.manousou@nhs.net
Jeremy F. Cobbold: jeremy.Cobbold@ouh.nhs.uk
Richard Parker: richardparker@nhs.net
David A Sheridan: david.sheridan2@nhs.net
Philip N Newsome: p.n.newsome@bham.ac.uk
Fredrik Karpe: fredrik.karpe@ocdem.ox.ac.uk
Matthew Neville: matthew.neville@ocdem.ox.ac.uk
Wiebke Arlt: w.arlt@lms.mrc.ac.uk
Alice J. Sitch: a.j.sitch@bham.ac.uk
Márta Korbonits: m.korbonits@qmul.ac.uk
Michael Biehl: m.biehl@rug.nl
William Alazawi: w.alazawi@qmul.ac.uk
Jeremy W Tomlinson: jeremy.tomlinson@ocdem.ox.ac.uk

Corresponding Author:
Prof Jeremy Tomlinson
Oxford Centre for Diabetes, Endocrinology & Metabolism
University of Oxford
Churchill Hospital
Headington
Oxford
OX3 7LJ
Email: jeremy.tomlinson@ocdem.ox.ac.uk
Tel. 01865 857359

For peer review only

Abstract

Introduction

Non-alcoholic fatty liver disease (NAFLD) affects approximately 1 in 4 individuals and its prevalence continues to rise. The advanced stages of NAFLD with significant liver fibrosis are associated with adverse morbidity and mortality outcomes. Currently, liver biopsy remains the ‘gold-standard’ approach to stage NAFLD severity. Although generally well tolerated, liver biopsies are associated with significant complications, are resource intensive, costly, sample only a very small area of the liver and require day case admission to a secondary care setting. As a result, there is a significant unmet need to develop non-invasive biomarkers that can accurately stage NAFLD and limit the need for liver biopsy. The aim of this study is to validate the use of the urine steroid metabolome as a strategy to stage NAFLD severity and to compare its performance against other non-invasive NAFLD biomarkers.

Methods and Analysis

The TrUSt NAFLD study is a multi-centre prospective test validation study aiming to recruit 310 patients with biopsy-proven and staged NAFLD across 8 centres within the UK. 150 appropriately matched control patients without liver disease will be recruited through the Oxford Biobank. Blood and urine samples, alongside clinical data, will be collected from all participants. Urine samples will be analysed by liquid chromatography-tandem mass spectroscopy to quantify a panel of pre-defined steroid metabolites. A machine learning based classifier, e.g. Generalized Matrix Relevance Learning Vector Quantization (GMLVQ) that was trained on retrospective samples, will be applied to the prospective steroid metabolite data to determine its ability to identify those patients with advanced, as opposed to mild-moderate, liver fibrosis as a consequence of NAFLD.

Ethics and dissemination

Research ethical approval was granted by West Midlands - Black Country Research Ethics Committee (REC reference: 21/WM/0177). A substantial amendment (TrUSt-NAFLD-SA1) was approved on 26/11/21.

Registration details

Title: Translating the potential of the urine steroid metabolome to stage non-alcoholic fatty liver disease

Trial ID: ISRCTN19370855

Date registered: 05/01/2023

For peer review only

Article summary

Strengths and limitations of this study:

Strengths

- The participants recruited with NAFLD patients will all have had a liver biopsy to stage their disease severity as well as multiple other non-invasive biomarkers that are currently used in clinical practice.
- The study includes a well-matched and phenotyped control cohort without evidence of liver disease, prospectively recruited from an existing biobank.
- A urinary test has high patient acceptability and is less operator dependent than other non-invasive tests such as transient elastography.

Limitations

- The study is cross-sectional and not longitudinal, so will not be able to address questions as to the utility of biomarker strategies and disease progression / remission.
- The study will recruit patients having a liver biopsy as part of routine clinical care which may introduce a degree of selection bias, creating a cohort more likely to have advanced (as opposed to mild) disease severity.

Keywords

Non-alcoholic fatty liver disease

urine steroid metabolome

biomarkers

For peer review only

Introduction

Non-alcoholic fatty liver disease (NAFLD) is the most prevalent chronic liver condition [1]. It affects up to 30% of the population (rising to 60% of patients with diabetes and obesity); >50 million adults in Europe and >10 million adults in the UK. It is the second commonest indication of liver transplantation worldwide [2, 3] and now accounts for 8.4% of liver transplants in Europe [4]. The advanced stages are associated with a 4-5-fold increase in mortality and morbidity, driven not only through liver-specific causes (including hepatocellular carcinoma), but through cardiovascular events [5, 6]. Estimates suggest that NAFLD costs the UK in excess of £5 billion per year and, globally, the expected 10-year burden of NAFLD could exceed $1.005 trillion in the USA and €334 billion in Europe [7].

NAFLD is a spectrum of disease ranging from simple steatosis through to inflammation (non-alcoholic steatohepatitis, NASH) to fibrosis (graded from F0-F4) with the risk of cirrhosis (F4) and the development of hepatocellular carcinoma (HCC). Fibrosis stage is tightly associated with an adverse clinical outcome with both liver-specific and cardiovascular morbidity and mortality [8, 9] The current reference standard for assessment of NAFLD severity is liver biopsy; however, there are significant complications and poor patient acceptability [10, 11]. In addition, it is a resource intensive and costly procedure, requires day case admission and time off work for the patient, samples a very small area of the liver and the interpretation of the biopsy findings are fraught with inter-individual variability [12]. Therefore, the development of accurate, non-invasive markers to stage NAFLD is a critical unmet priority clinical need. Routine interpretation of liver biochemistry is unhelpful; 50% of patients with advanced fibrosis (F3 or F4) have normal liver chemistry [13]. Several non-invasive tools (serological, clinical, imaging) have been developed; however, to date, none have been shown to be sufficiently robust to replace liver biopsy [14].

The liver is the main site of steroid metabolism and there is disruption of specific pathways in patients with NAFLD [15, 16], providing biological plausibility to our approach. The pilot study showed an increased activity in 11β-hydroxysteroid dehydrogenase type 1 (11BHSD1) and 5α-reductase in patients with advanced fibrosis [17]. Steroid 5β- reductase (AKR1D1), which is mostly expressed in the liver, has also been shown to decrease as fibrosis in NAFLD progresses [18]. When coupled with machine learning-based analysis using, for instance, Generalized Matrix Learning Vector Quantization (GMLVQ), a global assessment of hepatic steroid hormone metabolism can be made. GMLVQ is a supervised machine learning technique whereby an algorithm is trained using known datasets, and then subsequently applied to unknown samples. GMLVQ is able to simplify complex data into clusters of patients

For peer review only

who have a high risk of having advanced fibrotic NAFLD. Data from our published proof-of-principle study indicate that this is tightly influenced by NAFLD stage [17]. As part of this discovery study, we analysed urine samples from 227 subjects (121 with biopsy-proven NAFLD and 106 controls). GMLVQ analysis achieved excellent separation of early (F0-2) from advanced (F3-4) fibrosis (AUC ROC: 0.92 [0.91-0.94]). There was near perfect separation of healthy controls from patients with advanced fibrotic NAFLD and from those with NAFLD cirrhosis. Further refinement of the model incorporated the 10 most discriminatory steroids as well as patient age and BMI which has further enhanced diagnostic performance [17].

The aim of the current study is to prospectively validate our published findings in an independent cohort of patients undergoing routine liver biopsy for NAFLD and to compare the performance of the machine learning based urine steroid metabolome test against other non-invasive biomarker strategies that are currently used in clinical practice.

For peer review only

Methods and analysis

Design

TrUSt-NAFLD is a prospective, observational, non-interventional clinical study where recruitment and participation is designed to coincide with routine National Health Service (NHS) clinical care aiming to avoid the need for additional venepuncture, minimise inconvenience to participants and maximize recruitment potential. Occasionally, additional study specific visits for participants will be scheduled where this can facilitate recruitment or ensure that samples are collected within the pre-defined time limitations of the study (samples collected within 12-months of liver biopsy).

A dedicated control cohort of participants without evidence of liver disease will be prospectively recruited from an existing biobank of patients (Oxford Biobank (OBB), https://www.oxfordbiobank.org.uk) who have undergone detailed metabolic phenotyping and provided consent to be recalled by phenotype for additional clinical studies. These participants will not undergo liver biopsies but will have a comprehensive assessment of non-invasive NAFLD biomarkers measured as well as urine steroid metabolite analysis.

Setting and participants

The study will take place in secondary care hospital settings in 8 dedicated hepatology clinics across the UK. Patients (male or female), aged ≥18 years, who are attending or have attended an NHS clinic for the diagnosis and / or management of NAFLD and who are scheduled to have a liver biopsy or who have had a liver biopsy to stage NAFLD within the last 12 months will be approached to participate. Control participants without evidence of NAFLD will be recruited from the Oxford Biobank based at the Oxford Centre for Endocrinology, Diabetes and Metabolism, Churchill Hospital Oxford, UK.

Patient and public involvement

The study protocol and associated supporting documentation was reviewed by a dedicated patient and public involvement panel that is supported by the National Institute for Health and Care Research (NIHR) Oxford Biomedical Research Centre (BRC) and their feedback was incorporated into the final study documents. The panel will continue to be involved with dissemination activities following study completion.

Recruitment

The TrUSt-NAFLD study will recruit patients with a diagnosis of NAFLD from dedicated NHS hepatology clinics. The initial approach for participants to be part of the study will be made by

For peer review only

members of the team directly involved in their clinical care. In some situations, an alternative recruitment strategy whereby patients (who have consented to be approached for future research) are contacted having been identified from existing clinical databases may be employed. To ensure sufficient recruitment of patients, 8 centres across the UK will participate in the study. Interested potential participants will be seen during their routine NHS clinic visit by the research team to confirm eligibility. Those patients identified from existing databases will be contacted by letter and their eligibility will have been assessed prior to contact by lead clinicians and researchers within the dedicated hepatology team.

Recruitment of control participants without NAFLD will utilize the OBB; a biobank of >9000 volunteers in Oxfordshire, UK who have undergone extensive metabolic phenotyping and consented to be re-approached for clinical research. In order to try to ensure the absence of significant liver disease, individuals with a HOMA-IR (as a marker of global insulin resistance) https://www.dtu.ox.ac.uk/homacalculator/ in the lowest 25%, body mass index (BMI) <35kg/m^2 and fasting triglyceride levels <2 mmol/l will be invited to participate. HOMA-IR, triglycerides and BMI are all measured and recorded as part of the detailed OBB metabolic phenotyping and therefore can be used in a recall-by-phenotype strategy. Invitations to take part in the study will be sent with an aim of recruiting 60% male and 40% female participants to match the anticipated sex distribution in the NAFLD cohort which has been estimated from local liver biopsy data. Only individuals with normal liver biochemistry, and non-invasive serum markers (including the enhanced liver fibrosis panel, ELF) indicating low-risk of advanced NAFLD will be included in the study.

Screening and eligibility assessment

For potential participants with a diagnosis of NAFLD, screening will be undertaken by the clinical care team through direct confirmation of age, establishing that there is a diagnosis of NAFLD and that they either have a liver biopsy scheduled or have had a liver biopsy within the last 12-months. Potential participants will be provided with a letter of invitation and a patient information leaflet (PIL) either at the time of their routine NHS clinic visit or sent by post. For potential control participants without NAFLD, screening of the existing OBB database will be undertaken and a letter of invitation and PIL will be sent by members of the OBB research team.

Specific inclusion and exclusion criteria are detailed below. There will be no exceptions made regarding eligibility and each participant must satisfy all the approved inclusion and exclusion criteria of the protocol.

For peer review only

Inclusion Criteria

Participants with NAFLD

- Participant is willing and able to give informed consent for participation in the study.
- Patients with a diagnosis of NAFLD who are scheduled for a liver biopsy, or the patient has had a liver biopsy with a confirmed diagnosis of NAFLD within the last 12 months.
- Aged ≥18 years.

Control participants without evidence of NAFLD

- Participant is willing and able to give informed consent for participation in the study.
- Bottom 25th percentile of HOMA-IR in Oxford Biobank.
- Body mass index (BMI) <35kg/$m^{2.}$
- Fasting triglycerides<2.0mmol/L.
- Aged ≥18 years.

Exclusion Criteria

All participants

- Insufficient understanding of written and verbal English.
- The participant has not had sufficient time to read the PIL and understand the study requirements.
- Hepatic steatosis, inflammation, or fibrosis with a primary aetiology other than NAFLD.

Study procedures

All participants will personally sign and date the latest approved version of the informed consent form before any study specific procedures are performed. Written and verbal versions of the PIL and informed consent forms will be presented to the participants detailing the exact nature of the study, what it will involve for the participant, the implications and constraints of the protocol, and any risks involved in taking part. It will be clearly stated that the participant is free to withdraw from the study at any time for any reason without prejudice to their future care, without affecting their legal rights, and with no obligation to give the reason for withdrawal. The participant will be allowed as much time as possible (and this may be less

For peer review only

than 24-hours) to consider the information, and the opportunity to question the investigators, their general practitioner or other independent parties to decide whether they will participate in the study. Consent may be obtained, and the study procedures performed on the same day. The person who obtains the informed consent will be suitably qualified, experienced and have been authorised to do so by the chief/principal investigator. A copy of the signed informed consent form will be given to the participant. The original signed form will be retained at the study site. The study is not randomised. Participants will be enrolled as they are consented. At enrolment, all participants will be assigned a unique identification number in a sequential order.

For all participants, demographic data will be collected including age, sex, ethnicity, height, weight and calculated BMI. Details of the participants' clinical history will also be collected, including any relevant past medical history (smoking, type 2 diabetes, hypertension, cardiovascular disease and alcohol use) as well as details of concomitant prescribed medications. Patients with NAFLD will usually have routine blood tests as part of their clinical care consultation and therefore the additional blood samples that will be collected will not require additional venepuncture (although this may be needed if a participant attends for a dedicated research visit that is separate from their clinical care appointment). A dedicated research visit is required for all participants without NAFLD that will include collecting demographic data and clinical history as well as venepuncture and providing a 10mL urine sample. Vibration-controlled transient elastography (VCTE) is frequently used in hepatology clinics to determine the risk of advanced fibrosis and can provide both a measure of liver stiffness (indicative of fibrosis stage) and a controlled attenuation parameter (CAP) that correlates with the degree of hepatic steatosis [19, 20]. Where available, these data will be recorded for all patients with NAFLD.

All blood sample analysis will include measurement of a full blood count, renal function and electrolytes, liver chemistry (including alanine aminotransaminase (ALT), aspartate aminotransaminase (AST), alkaline phosphatase (ALP), gamma-glutamyltransferase (GGT), bilirubin, albumin, ferritin), total and HDL-cholesterol and triglycerides as well as glycated haemoglobin. In addition, samples will be analysed for the Enhanced Liver Fibrosis (ELF) panel [21] (hyaluronic acid, type III procollagen peptide, and tissue inhibitor of matrix metalloproteinase 1). Calculations for non-invasive risk score to detect advanced fibrosis will also be made including NAFLD fibrosis score (NFS), Fib-4, APRI, Fibroscan-AST (FAST) and BARD [22-26].

Liver biopsy data will be collected to include the NAFLD activity score (NAS) grading with its individual components of steatosis, lobular inflammation, and ballooning and also the fibrosis staging (graded F0-F4) according to the Kleiner classification [27].

For peer review only

A 10mL urine sample will be collected from all participants, aliquoted and stored at -80°C until analysis. Pre-defined steroid hormone metabolites will be measured using gas chromatography mass spectrometry (GC-MS) and liquid chromatography tandem mass spectrometry (LC MS-MS) as described previously [28] [29]. The steroid metabolite data will then be subjected to machine learning employing a GMLVQ [17] classifier that was trained on retrospective data. Its performance to identify participants with advanced fibrotic NAFLD compared against other non-invasive biomarker strategies (Fib-4, NFS, BARD, APRI, AST/ALT, ELF, FAST-AST, VCTE) will be evaluated.

Statistical methods, sample size and analysis plan

The study has been planned and designed with expert statistical advice and an experienced statistician is part of the investigative team.

Sample size

We will aim to recruit at least 460 participants to the TrUSt-NAFLD study (NAFLD n=310; control n=150). Sample size and power calculations have been based upon our previous published data [17]. Our primary analysis will aim to distinguish patients with mild (F0-2) *vs.* advanced fibrosis (F3-4). Through the recruitment of 310 participants with NAFLD, we will have 85% power to identify a difference in sensitivity between technologies of 12 percentage points (80% vs 92%) assuming a 5% significance level, a prevalence of 50% (i.e. 50% of the participants will be F0-2 and 50% F3-4) and independence of comparative test results. The estimates of sensitivity and the difference in sensitivity have been estimated from previous work [14].

Analysis of Outcome Measures

Data for all participants who complete the study will be included in the analysis. If any participants withdraw from the study, samples and data collected will not routinely be destroyed and will be used in the analysis unless requested not to do so by the individual. Statistical analysis will be undertaken at the end of study and after the analysis of all the samples has been completed.

The primary outcome of the TrUSt-NAFLD study is to determine the accuracy of the urine steroid metabolome GMLVQ algorithm to distinguish early (F0-2) from advanced (F3-4) NAFLD fibrosis. Sensitivity, specificity, negative and positive predictive values will be

For peer review only

calculated with 95% confidence intervals. Additionally, receiver operating characteristic curves will be generated and the area under the curve will be calculated.

Secondary and exploratory outcomes will include a comparison of the performance of the urine steroid metabolome GMLVQ algorithm against other non-invasive NAFLD biomarkers (Fib-4, NFS, BARD, APRI, AST/ALT, ELF, FAST-AST, VCTE). Data from participants with NAFLD will also be compared against control participants without evidence of NAFLD to determine the ability of the algorithm to detect NAFLD (across the spectrum of its severity) in the general population. Finally, exploratory analyses detecting steroid metabolites using LC MS-MS in serum samples and subsequently incorporating the GMLVQ algorithm will be performed and compared against the urine analysis.

Discussion:

This diagnostic test using the urinary steroid metabolome represents a unique way to diagnose and stage NAFLD without the need for blood tests, biopsy or imaging. It has shown promising results in a published pilot study and the TrUSt NAFLD study aims to validate these findings in a larger, prospectively collected cohort. Urine steroid metabolites are measured using liquid chromatography tandem mass spectrometry before applying machine learning techniques including GMLVQ. During the pilot study, prototype urine steroid metabolome profiles for each fibrosis stage were created using GMLVQ. When the algorithm is presented with an unknown sample, the dissimilarity between the unknown urine steroid profile and the profile of established prototype is measured. Based on this, the sample is then assigned a disease stage based upon the closest similarity to a specific prototype. Both the liquid chromatography tandem mass spectrometry and machine learning analysis represent potential drawbacks of this study given their relative complexity. In addition, such an analytical capability may not be currently available at all clinical centres. However, liquid chromatography tandem mass spectrometry is a relatively high throughput approach where samples can be analysed rapidly. A strength of the study is that the participants with NAFLD have biopsy-proven and histologically staged disease. However, within the control cohort, it is neither realistic nor ethically acceptable to undertake a liver biopsy (bearing in mind the associated morbidity and mortality), but this serves to emphasise the importance of developing non-invasive tests with good accuracy alongside high patient acceptability. Should the data from this study validate our approach, then there is the realistic potential for major clinical impact, changing how NAFLD is diagnosed and staged.

For peer review only

Ethics and dissemination

Ethical and safety considerations

The chief investigator will ensure that this study is conducted in accordance with the principles of the Declaration of Helsinki and that the study is conducted in accordance with relevant regulations and with Good Clinical Practice.

The study will not pose significant ethical issues or risks to the participants. Sample collection and study visits for those individuals with NAFLD will, in the majority of cases, coincide with routine clinical care appointments, and so additional venepuncture, and its associated discomfort, will not be needed. Providing the urine sample provides no additional risk. Where additional study appointments are required, travel expenses are reimbursed and a small payment to cover inconvenience will be offered. Incidental abnormal finding unrelated to the study will be discussed with the participants and where appropriate, and with their consent, referral to their general practitioner or suitable specialist will be made.

The TrUSt-NAFLD study will be sponsored by University of Oxford, UK. All relevant study supporting documentation as well as the clinical protocol have been reviewed and approved by West Midlands, Black Country Research Ethics Committee (REC) (ref. 21/WM/0177) and the Health Research Authority (HRA) (https://www.hra.nhs.uk).

The OBB is sponsored by the University of Oxford, UK and supported by the NIHR Oxford BRC. The protocols and supporting documentation for recruitment, investigations and recruiting by phenotype and genotype have been reviewed and approved by South Central, Oxford C NHS REC (ref. 18/SC/0588) and the HRA. An annual progress report will be submitted to the REC and HRA (where required) host organisation and sponsor. In addition, on study completion, an End of Study notification and final report will be submitted to the same parties.

Transparency in research

Details of the study will be made available on HRA research summaries https://www.hra.nhs.uk/planning-and-improving-research/application-summaries/research-summaries/. The study details will also be available on University of Oxford and Radcliffe Department of Medicine websites. The study is registered on the International Standard Registered Clinical/soCial sTudy Number (ISRCTN) database (ISRCTN19370855, https://doi.org/10.1186/ISRCTN19370855).

For peer review only

Data statement

The datasets generated during and/or analysed during the current study will be stored in a non-publicly available repository

Dissemination plan (publications, data deposition and curation)

A copy of the final report and / or publication will be sent to study participants upon request. Results will be disseminated through presentations at national and international conferences and publications sent to peer-review journals with open access in accordance with the funder's publication policy (https://wellcome.org/grant-funding/guidance/open-access-guidance/open-access-policy). In addition, research findings will be disseminated through dedicated departmental public engagement events and through patient support groups.

The investigators will be involved in reviewing drafts of the manuscripts, abstracts, press releases and any other publications arising from the study. Authors will acknowledge that the study was funded by Wellcome. Authorship will be determined in accordance with the ICMJE guidelines and other contributors will be acknowledged.

For peer review only

Author contributions

HM will co-ordinate and run the TrUSt NAFLD study and drafted the manuscript. JWT is the chief investigator for the study and was responsible for the original concept and design of the study. JWT edited the manuscript and was responsible for the final approval of the manuscript.

DH, GA, PM, JC, RP, DS, PN, FK, MN, WA, AS, MK, MB, WA and JWT were all involved in securing funding, they have all reviewed the protocol and are named investigators and they have all contributed to the writing and reviewing of the manuscript.

Funding statement

This study is funded through a Wellcome Innovator Award to JWT, MB and WA (ref: 222627/Z/21/Z) and is supported by the NIHR Oxford Biomedical Research Centre and by the NIHR Birmingham Biomedical Research Centre. The views expressed are those of the authors and not necessarily those of the NIHR or the Department of Health and Social Care.

Competing interest statement

JWT has been an advisory board member for Novo Nordisk, Pfizer and Poxel and has been part of a data and safety monitoring committee for Novartis. RP has been an advisory board member for Novo Nordisk.

A patent related to the proposed work has been filed (GB Patent Application Number 2005435.9, filed 14/4/20, European Patent Application Number: EP21703541.9A, filed 29/1/21 and Worldwide Patent Application Number: WO2021152330A1, filed 29/1/21)

Word count = 3501

For peer review only

References

1. Paik, J.M., et al., *Changes in the Global Burden of Chronic Liver Diseases From 2012 to 2017: The Growing Impact of NAFLD.* Hepatology, 2020. **72**(5): p. 1605-1616.
2. Wong, R.J., et al., *Nonalcoholic steatohepatitis is the second leading etiology of liver disease among adults awaiting liver transplantation in the United States.* Gastroenterology, 2015. **148**(3): p. 547-55.
3. Dulai, P.S., et al., *Increased risk of mortality by fibrosis stage in nonalcoholic fatty liver disease: Systematic review and meta-analysis.* Hepatology, 2017. **65**(5): p. 1557-1565.
4. Haldar, D., et al., *Outcomes of liver transplantation for non-alcoholic steatohepatitis: A European Liver Transplant Registry study.* J Hepatol, 2019. **71**(2): p. 313-322.
5. Singal, A.G., P. Lampertico, and P. Nahon, *Epidemiology and surveillance for hepatocellular carcinoma: New trends.* J Hepatol, 2020. **72**(2): p. 250-261.
6. Younossi, Z.M., et al., *Global epidemiology of nonalcoholic fatty liver disease-Meta-analytic assessment of prevalence, incidence, and outcomes.* Hepatology, 2016. **64**(1): p. 73-84.
7. Younossi, Z.M., et al., *The economic and clinical burden of nonalcoholic fatty liver disease in the United States and Europe.* Hepatology, 2016. **64**(5): p. 1577-1586.
8. Taylor, R.S., et al., *Association Between Fibrosis Stage and Outcomes of Patients With Nonalcoholic Fatty Liver Disease: A Systematic Review and Meta-Analysis.* Gastroenterology, 2020. **158**(6): p. 1611-1625.e12.
9. Sanyal, A.J., et al., *Prospective Study of Outcomes in Adults with Nonalcoholic Fatty Liver Disease.* New England Journal of Medicine, 2021. **385**(17): p. 1559-1569.
10. Neuberger, J., et al., *Guidelines on the use of liver biopsy in clinical practice from the British Society of Gastroenterology, the Royal College of Radiologists and the Royal College of Pathology.* Gut, 2020. **69**(8): p. 1382.
11. Howlett, D.C., et al., *Findings of the UK national audit evaluating image-guided or image-assisted liver biopsy. Part II. Minor and major complications and procedure-related mortality.* Radiology, 2013. **266**(1): p. 226-35.
12. Brunt, E.M., et al., *Complexity of ballooned hepatocyte feature recognition: Defining a training atlas for artificial intelligence-based imaging in NAFLD.* Journal of Hepatology, 2022. **76**(5): p. 1030-1041.
13. Verma, S., et al., *Predictive value of ALT levels for non-alcoholic steatohepatitis (NASH) and advanced fibrosis in non-alcoholic fatty liver disease (NAFLD).* Liver International, 2013. **33**(9): p. 1398-1405.
14. Chin, J.L., et al., *Non-invasive Markers of Liver Fibrosis: Adjuncts or Alternatives to Liver Biopsy?* Front Pharmacol, 2016. **7**: p. 159.
15. Ahmed, A., et al., *A Switch in Hepatic Cortisol Metabolism across the Spectrum of Non Alcoholic Fatty Liver Disease.* PLoS ONE, 2012. **7**(2): p. e29531.
16. Nikolaou, N., et al., *AKR1D1 is a novel regulator of metabolic phenotype in human hepatocytes and is dysregulated in non-alcoholic fatty liver disease.* Metabolism, 2019. **99**: p. 67-80.
17. Moolla, A., et al., *Accurate non-invasive diagnosis and staging of non-alcoholic fatty liver disease using the urinary steroid metabolome.* Alimentary Pharmacology & Therapeutics, 2020. **51**(11): p. 1188-1197.

For peer review only

18. Nikolaou, N., et al., *AKR1D1 is a novel regulator of metabolic phenotype in human hepatocytes and is dysregulated in non-alcoholic fatty liver disease.* Metabolism, 2019. **99**: p. 67-80.
19. Siddiqui, M.S., et al., *Vibration-Controlled Transient Elastography to Assess Fibrosis and Steatosis in Patients With Nonalcoholic Fatty Liver Disease.* Clinical Gastroenterology and Hepatology, 2019. **17**(1): p. 156-163.e2.
20. Pu, K., et al., *Diagnostic accuracy of controlled attenuation parameter (CAP) as a non-invasive test for steatosis in suspected non-alcoholic fatty liver disease: a systematic review and meta-analysis.* BMC Gastroenterology, 2019. **19**(1).
21. Guha, I.N., et al., *Noninvasive markers of fibrosis in nonalcoholic fatty liver disease: Validating the European Liver Fibrosis Panel and exploring simple markers.* Hepatology, 2007. **47**(2): p. 455-460.
22. Angulo, P., et al., *The NAFLD fibrosis score: A noninvasive system that identifies liver fibrosis in patients with NAFLD.* Hepatology, 2007. **45**(4): p. 846-854.
23. Sterling, R.K., et al., *Development of a simple noninvasive index to predict significant fibrosis in patients with HIV/HCV coinfection.* Hepatology, 2006. **43**(6): p. 1317-1325.
24. Wai, C., *A simple noninvasive index can predict both significant fibrosis and cirrhosis in patients with chronic hepatitis C.* Hepatology, 2003. **38**(2): p. 518-526.
25. Newsome, P.N., et al., *FibroScan-AST (FAST) score for the non-invasive identification of patients with non-alcoholic steatohepatitis with significant activity and fibrosis: a prospective derivation and global validation study.* The Lancet Gastroenterology and Hepatology, 2020. **5(4)**: p. 362-373.
26. Harrison, S.A., et al., *Development and validation of a simple NAFLD clinical scoring system for identifying patients without advanced disease.* Gut, 2008. **57**(10): p. 1441-7.
27. Kleiner, D.E., et al., *Design and validation of a histological scoring system for nonalcoholic fatty liver disease.* Hepatology, 2005. **41**(6): p. 1313-1321.
28. Arlt, W., et al., *Urine Steroid Metabolomics as a Biomarker Tool for Detecting Malignancy in Adrenal Tumors.* The Journal of Clinical Endocrinology & Metabolism, 2011. **96**(12): p. 3775-3784.
29. Bancos, I., et al., *Urine steroid metabolomics for the differential diagnosis of adrenal incidentalomas in the EURINE-ACT study: a prospective test validation study.* Lancet Diabetes Endocrinol, 2020. **8**(9): p. 773-781.

For peer review only
